# Experimental Inoculation of Young Calves with SARS-CoV-2

**DOI:** 10.3390/v13030441

**Published:** 2021-03-09

**Authors:** Shollie Falkenberg, Alexandra Buckley, Melissa Laverack, Mathias Martins, Mitchell V. Palmer, Kelly Lager, Diego G. Diel

**Affiliations:** 1 Ruminant Disease and Immunology Research Unit, National Animal Disease Center, USDA, Agricultural Research Service, 1920 Dayton Avenue, P.O. Box 70, Ames, IA 50010, USA; 2 Virus and Prion Research Unit, National Animal Disease Center, USDA, Agricultural Research Service, 1920 Dayton Avenue, P.O. Box 70, Ames, IA 50010, USA; alexandra.buckley@usda.gov (A.B.); kelly.lager@usda.gov (K.L.); 3 Department of Population Medicine and Diagnostic Sciences, Animal Health Diagnostic Center, College of Veterinary Medicine, Cornell University, 240 Farrier Rd, Ithaca, NY 14853, USA; mp75@cornell.edu (M.L.); mm3245@cornell.edu (M.M.); dgdiel@cornell.edu (D.G.D.); 4 Infectious Bacterial Diseases Research Unit, National Animal Disease Center, USDA, Agricultural Research Service, 1920 Dayton Avenue, P.O. Box 70, Ames, IA 50010, USA; mitchell.palmer@usda.gov

**Keywords:** bovine, inoculation, SARS-CoV-2

## Abstract

The host range of SARS-CoV-2 and the susceptibility of animal species to the virus are topics of great interest to the international scientific community. The angiotensin I converting enzyme 2 (ACE2) protein is the major receptor for the virus, and sequence and structural analysis of the protein has been performed to determine its cross-species conservation. Based on these analyses, cattle have been implicated as a potential susceptible species to SARS-CoV-2 and have been reported to have increased ACE2 receptor distribution in the liver and kidney, and lower levels in the lungs. The goal of the current study was to determine the susceptibility of cattle to SARS-CoV-2 utilizing inoculation routes that facilitated exposure to tissues with increased ACE2 receptor distribution. For this, colostrum-deprived calves approximately 6 weeks of age were inoculated via the intratracheal or intravenous routes. Nasal and rectal swab samples, as well as blood and urine samples, were collected over the course of the study to evaluate viral shedding, viremia, and seroconversion. Pyrexia was used as the primary criteria for euthanasia and tissue samples were collected during necropsy. Importantly, SARS-CoV-2 RNA was detected in only two nasal swab samples collected on days 3 and 10 post-inoculation (pi) in two calves; one calf in the intratracheal group and the other calf in the intravenous group, respectively. Additionally, the calf in the intratracheal group that was positive on the nasal swab on day 3 pi also had a positive tracheobronchial lymph node on day 9 pi. Viral nucleic acid load on these samples, based on PCR cycle threshold values, were low and infectious virus was not recovered from the samples. These results suggest that there was no productive replication of SARS-CoV-2 in calves following intratracheal and intravenous inoculation.

## 1. Introduction

Severe acute respiratory syndrome coronavirus 2 (SARS-CoV-2) is a novel coronavirus, within the family Coronaviridae, genus Betacoronavirus (subgenus Sarbecovirus) that causes coronavirus disease 19 (COVID-19) in humans [1]. SARS-CoV-2 was first reported in Wuhan, Hubei province, China in December 2019 [2]. The first reported cases had an epidemiological link to the Huanan Seafood Wholesale market in Hubei where several live animal species were sold [2,3,4].

Given the presumed zoonotic origin of SARS-CoV-2, and the potential involvement of an intermediate animal host in the transmission of the virus to humans [3,4,5,6,7,8], the susceptibility of animals to SARS-CoV-2 and investigations on their potential role as reservoirs of SARS-CoV-2 have received significant interest worldwide. Coronaviruses are known for their ability to cross the species barrier and establish new infection hosts, including humans. For example, before the emergence of SARS-CoV in 2002–2003, two other coronaviruses (CoV) of zoonotic origin had been reported in humans, including HCoV-229E, an alphacoronavirus originating in bats and passing to humans through alpacas, and HCoV-OC43, a betacoronavirus which originated in rodents and was transmitted to humans through cattle [9]. Importantly, another ruminant species, dromedary camels were considered the intermediate host of MERS-CoV, which emerged in humans in the Middle East in 2012 [9]. Therefore, ruminant species have played an important role in the spread of coronavirus in the past and served as intermediate reservoirs to humans.

The host-range of SARS-CoV-2 is thought to be largely dependent on the interaction of the virus receptor binding protein—the Spike protein—with the host cell receptor—angiotensin I converting enzyme 2 (ACE2) [5,10,11,12]. Given the potential for interspecies transmission, several studies have recently focused on predictive analyses of the binding potential of SARS-CoV-2 with the ACE2 receptor in different animal species [11,13,14]. These studies predicted that several mammalian species, including important domestic livestock and wildlife ruminant species, could potentially be susceptible to SARS-CoV-2 infection. Notably, we have recently demonstrated that white-tailed deer fawns are susceptible to SARS-CoV-2 infection [15], confirming in silico predictions that ranked this species in the high risk category [14].

Natural infections with SARS-CoV-2 have been reported in a variety of animal species including dogs, cats, farmed mink, tigers, and lions in Hong Kong, Netherlands, China, and the United States [7,16,17,18]. Additionally, experimental inoculation demonstrated the susceptibility of non-human primates, ferrets, minks, cats, dogs, raccoon dogs, golden Syrian hamsters, deer mice, and white-tailed deer as evidenced by mild to moderate clinical disease and/or sustained viral replication, suggesting a productive infection after inoculation with SARS-CoV-2 [15,19,20,21,22,23,24,25]. Whereas experimental inoculation of swine, cattle, poultry, and fruit bats have shown that these species are either not susceptible to SARS-CoV-2 or that inoculation did not result in productive infection and sustained viral replication [26,27,28,29,30].

Initial in vivo studies utilizing intranasal inoculation in cattle suggested a lack of sustained viral replication, as evidenced mostly by lack of viral detection by RT-PCR with only a few samples from two animals being detected over the course of the study [26]. Although, ex vivo organ cultures from ruminants have been shown susceptible, demonstrating sustained viral replication and an association of SARS-CoV-2 with ACE2-expressing cells [31]. Interestingly, the distribution of the ACE2 receptor in cattle has been reported to be higher in the liver and kidney, with the lung only having moderate ACE2 receptor expression compared to other tissues [32].

Given the susceptibility of ruminants to other betacoronaviruses and the predictive analysis suggestive of binding potential for SARS-CoV-2 in ruminants including cattle, in the present study we investigated the susceptibility of cattle to SARS-CoV-2 following intratracheal and intravenous inoculation.

## 2. Methods

### 2.1. Experimental Design and Sample Collection

All animals were handled in accordance with the Animal Welfare Act Amendments (7 U.S. Code §2131 to §2156) and all study procedures were reviewed and approved by the Institutional Animal Care and Use Committee at the National Animal Disease Center (ARS-2020-861).

Six colostrum-deprived Holstein bull calves were purchased at approximately 6 weeks of age from a commercial vendor. Prior to arrival, all calves were tested to ensure they were negative for BVDV antigen and antibodies as described previously [33]. Upon arrival, the calves were moved into the same isolation room in a biosafety level 3 (Agriculture) (BSL-3Ag) biocontainment facility. The room was divided using a plexiglass barrier approximately 1 m in height to prevent nose-to-nose contact and separate the two treatment groups. Airflow in the room was maintained at 10–11 air exchanges per hour, at a standard exchange rate for ABSL-3 housing of large animals.

Upon arrival, a rumen temperature probe given as a bolus to record basal temperatures was administered as previously described [34]. At −2 days post-inoculation (pi), calves were started on probiotics and remained on the probiotic regime for the duration of the study. A prophylactic antibiotic (Baytril^®^; 5 mL subcutaneously) and coccidiostat (Corid^®^; 5 mL orally in milk replacer for 5 days) treatment was initiated at the same time for preventive measures.

Sample time points included three baseline samples collected on days −5, −4, and 0 pi. These baseline samples were used to confirm negative antibody and antigen status to other bovine respiratory pathogens as well as SARS-CoV-2. On day 0 pi, three calves were inoculated with the SARS-CoV-2 strain, TGR1/NY/20 [7] via the intratracheal (IT) or intravenous (IV) routes (5 mL each respective route). Samples were collected on days 2, 3, 4, 5, 6, 7, 8, 9, 10, 12, 14, and 21 pi and included nasal and rectal swabs, whole blood, and voided urine (when possible) for virus detection. Serum was obtained during baseline sample days and at days 7, 14, and 21 pi and processed for virus neutralization assays [7]. One calf from each group was humanely euthanized by IV administration of sodium pentobarbital on days 9, 16, and 21 pi for necropsy with pyrexia used as the primary criteria for euthanasia. Following necropsy, multiple specimens including tracheal wash (TW), lung lavage (LL), and several tissues (nasal turbinates, palatine tonsil, thymus, trachea, lung, bronchi, kidney, liver, spleen, ileum, ileocecal junction, spiral colon, cerebellum, cerebrum, olfactory bulbs and medial retropharyngeal, mandibular, tracheobronchial, mediastinal and mesenteric lymph nodes) were collected. Samples were processed for real-time reverse transcriptase PCR (rRT-PCR) and virus isolation (VI) and were individually bagged, placed on dry ice, and transferred to a −80 °C freezer until testing. Additionally, tissue samples were collected and processed for standard microscopic examination and in situ hybridization (ISH). For this, tissue fragments of approximately ≤0.5 cm in width were fixed by immersion in 10% neutral buffered formalin (≥20 volumes fixative to 1 volume tissue) for approximately 24 h, and then transferred to 70% ethanol, followed by standard paraffin embedding techniques. Slides for standard microscopic examination were stained with hematoxylin and eosin (HE).

### 2.2. Assessing the Presence or Exposure to Other Bovine Respiratory Pathogens

Nasal swabs, lung lavage, and serum collected at both baseline and necropsy timepoints for each calf were assessed for the presence and exposure to other bovine pathogens. Nasal swabs and lung lavage samples were submitted to the Iowa State Veterinary Diagnostic Laboratory for complete bovine respiratory viral and bacterial PCR panels to detect the presence of bovine herpesvirus-1 (BoHV-1), bovine coronavirus (BCoV), bovine viral diarrhea virus (BVDV), bovine respiratory syncytial virus (BRSV), *Pasteurella multocida*, *Mycoplasma bovis*, *Mannheimia haemolytica*, and *Histophilus somni*. Both baseline and necropsy serum samples were tested using a commercial ELISA (BioX ELISA; BioX Diagnostics, Rochefort, Belgium, Europe) for detection of antibodies for multiple viral pathogens that include; BVDV, BRSV, BoHV-1, bovine parainfluenza virus-3 (BPI-3), and bovine adenovirus-3 (BADV-3). Detection of BCoV antibodies were performed as previously described [35].

### 2.3. Cells and Virus

Vero cells (ATCC^®^ CCL-81™, American Type Culture Collection, Manassas, VA, USA), Vero E6/TMPRSS2 (JCRB1819, Japanese Cancer Research Resources [JCRB] Bank, Ibaraki city, Osaka, Japan), and bovine turbinate (BT) cells were cultured in Dulbecco’s modified Eagle medium (DMEM), while *Bos taurus* trachea normal (EBTr (NBL-4)), cow pulmonary artery epithelial (CPAE), primary fetal bovine lung (FBL), and fetal bovine kidney (FBK) cells were cultured in minimum essential medium (MEM). Both DMEM and MEM were supplemented with 10% fetal bovine serum (FBS), L-glutamine (2 mM), penicillin (100 U·mL^−1^), streptomycin (100 μg·mL^−1^), and gentamycin (50 μg·mL^−1^). The cell cultures were maintained at 37 °C with 5% CO_2_. The SARS-CoV-2 isolate TGR/NY/20 obtained from a Malayan tiger naturally infected with SARS-CoV-2 and presenting with respiratory disease compatible with SARS-CoV-2 infection [7] was propagated in Vero cells. It is important to note that the TGR/NY/20 virus detected in the Malayan tiger was the first detection of human to animal transmission of SARS-CoV-2 in the US and the virus is identical to human SARS-CoV-2 strains and contains the D614G mutation. Low passage virus stocks (passage 4) were prepared, cleared by centrifugation (1966× *g* for 10 min), and stored at −80 °C. The endpoint titer was determined by limiting dilution following the Spearman and Karber method. A viral suspension containing 6.8 × 10^6^ TCID_50_.mL^−1^ was obtained and used for animal inoculation.

### 2.4. Cell Susceptibility

The susceptibility of bovine cells to SARS-CoV-2 was assessed in vitro and compared to virus replication in Vero E6/TMPRSS2. For this, BT, EBTr (NBL-4), CPAE, FBL, and FBK, as well as Vero E6/TMPRSS2 cells, were inoculated with SARS-CoV-2 isolate TGR/NY/20 at a multiplicity of infection of 1 or 0.1 (MOI = 1 or 0.1) and the cells incubated for 24 h at 37 °C with 5% CO_2_. At 72 h post-inoculation, cells were fixed with 3.7% formaldehyde for 30 min at room temperature (RT), permeabilized with 0.2% Triton X-100 (in phosphate buffered saline (PBS)) for 10 min at RT and subjected to an immunofluorescence assay (IFA) using a monoclonal antibody (MAb) anti-SARS-CoV-2 nucleoprotein (N) (clone B6G11) produced and characterized in Dr. Diel’s laboratory, and then incubated with a goat anti-mouse IgG secondary antibody (goat anti-mouse IgG, Alexa Fluor^®^ 594), and Nuclear counterstain was performed with DAPI, and visualized under a fluorescence microscope.

### 2.5. RNA Extraction and Real-Time RT-PCR (RT-rPCR)

For virus detection, jugular vein blood samples were collected in EDTA tubes, and buffy coat (BC) was separated by centrifugation (25 min at 1200× *g*). The BC was collected and subjected to one freeze/thaw cycle (−80 °C). An aliquot of 200 µL of BC sample was used for RNA extraction using the MagMax Core extraction kit (Thermo Fisher, Waltham, MA, USA) and the automated KingFisher Flex nucleic acid extractor (Thermo Fisher, Waltham, MA, USA) following the manufacturer’s recommendations.

Upon collection, each respective nasal swab (NS) and rectal swab (RS) sample was placed in a 5 mL snap cap tube pre-filled with 2 mL of viral transport media (MEM with anti/anti). NS and RS samples were stored at −80 °C until analysis. After thawing, the NS samples containing the swab were centrifuged for 2 min at 400× *g* to separate any debris from the swab itself. Similar to the BC sample, an aliquot of 200 µL of the NS and RS sample was submitted to RNA extraction as stated previously. The rRT-PCR) was performed using the EZ-SARS-CoV-2 Real-Time RT-PCR assay (Tetracore Inc., Rockville, MD, USA). An internal inhibition control was included in all reactions. Positive and negative amplification controls were run side-by-side with test samples. All RNA extractions and rRT-PCR were performed at the Cornell Animal Health Diagnostic Center (AHDC).

Urine was clean caught in a 15 mL conical tube at each sample collection, and urine samples were stored at −80 °C until analysis. After thawing, 200 µL of each urine sample was used for RNA extraction and rRT-PCR as described above.

Tissue samples collected at necropsy were stored at −80 °C until analysis. After thawing, 0.5 g of tissues were minced with a sterile scalpel and resuspended in 5 mL MEM. Samples were cleared by centrifugation and 200 µL of the cleared supernatant was used for RNA extraction and rRT-PCR testing as described above.

### 2.6. Virus Isolation

Any samples that tested positive for SARS-CoV-2 by rRT-PCR were subjected to virus isolation under biosafety level 3 (BSL-3) conditions at Cornell AHDC. Twenty-four well plates were seeded with ~75,000 Vero E6/TMPRSS2 cells per well 24 h prior to sample inoculation. Cells were rinsed with phosphate buffered saline (PBS) (Corning^®^) and inoculated with 150 μL of each sample and inoculum adsorbed for 1 h at 37 °C with 5% CO_2_. Mock-inoculated cells were used as negative controls. After adsorption, replacement cell culture media supplemented as described above was added, and cells were incubated at 37 °C with 5% CO_2_ and monitored daily for cytopathic effect (CPE) for 3 days. SARS-CoV-2 infection in CPE-positive cultures was confirmed with an immunofluorescence assay (IFA) as described above. Cell cultures with no CPE were frozen, thawed, and subjected to two additional blind passages/inoculations in Vero E6/TMPRSS2 cell cultures as described above. At the end of the third passage, the cells cultures were subjected to IFA as above.

### 2.7. Serological Analysis

Blood was collected into serum separation tubes with gel and clot activator (BD Vacutainer SST, Franklin Lakes, NJ, USA), centrifuged (25 min at 1200× *g*), and serum was collected and frozen at −80 °C until testing. Neutralizing antibodies to SARS-CoV-2 was assessed by a virus neutralization assay (VNA) performed under BSL-3 conditions at the Cornell AHDC. Twofold serial dilutions (1:4 to 1:4096) of serum samples were incubated with 100–200 TCID50 of SARS-CoV-2 isolate TGR/NY/20 for 1 h at 37 °C. Following incubation of serum and virus, 50 μL of a cell suspension of Vero cells was added to each well of a 96-well plate and incubated for 48 h at 37 °C with 5% CO_2_. The cells were fixed and permeabilized as described above and subjected to IFA using a rabbit polyclonal antibody (pAb) specific for the SARS-CoV-2 nucleoprotein (N) (produced in Dr. Diel’s laboratory), followed by incubation with a goat anti-rabbit IgG (goat anti-rabbit IgG, DyLight^®^ 594 Conjugate, Immunoreagent Inc. Raleigh, NC, USA). Unbound antibodies were washed from cell cultures by rinsing the cells in PBS, and virus infectivity was assessed under a fluorescence microscope. Neutralizing antibody titers were expressed as the reciprocal of the highest dilution of serum that completely inhibited SARS-CoV-2 infection/replication. Fetal bovine serum (FBS) and convalescent human serum (kindly provided by Dr. Elizabeth Plocharczyk, Cayuga Medical Center (CMC), under CMC’s Institutional Review Board protocol number 0420EP) were used as negative and positive controls, respectively.

### 2.8. In Situ Hybridization (ISH)

Paraffin-embedded tissues were sectioned at 5 μm and subjected to ISH using the RNAscope ZZ probe technology (Advanced Cell Diagnostics, Newark, CA, USA). In situ hybridization was performed to detect tissue distribution of SARS-CoV-2 nucleic acid in palatine tonsil, tracheobronchial-lymph nodes, nasal turbinate, lung, trachea, and kidney, using the RNAscope 2.5 HD Reagents–RED kit (Advanced Cell Diagnostics, Newark, CA, USA) as previously described (67). Proprietary ZZ probes targeting SARS-CoV-2 RNA (V-nCoV2019-S probe ref# 8485561) designed and manufactured by Advance Cell Diagnostics were used for detection of viral RNA. A positive control probe targeted the *Bos taurus*-specific cyclophilin B (PPIB Cat# 3194510) or ubiquitin (UBC Cat # 464851) housekeeping genes, while a probe targeting dapB of *Bacillus subtilis* (Cat # 312038) was used as a negative control.

In situ hybridization was performed on the turbinates, palatine tonsil, lung, and kidney to detect tissue distribution of mRNA ACE2 receptor distribution using the BaseScope 2.5 HD Reagents–RED kit (Advanced Cell Diagnostics, Newark, CA, USA) as previously described [15]. Proprietary ZZ probes targeting the region spanning AA 31–82 for the ACE2 receptor specific to *Bos taurus* (BA-Bt-ACE2-1zz-st probe; ref# 901071) designed and manufactured by Advance Cell Diagnostics. The slides were counterstained with hematoxylin and examined by light microscopy using a Nikon Eclipse Ci microscope. Digital images were captured using a Nikon DE-Ri2 camera.

## 3. Results

### 3.1. Assessing In Vitro Replication of SARS-CoV-2 in Bovine Cells

The ability of SARS-COV-2 to infect and replicate in bovine cells was assessed in vitro. For this, bovine turbinate (BT), *Bos taurus* trachea normal (EBTr (NBL-4)), cow pulmonary artery epithelial (CPAE), primary fetal bovine lung (FBL), and fetal bovine kidney (FBK) cells were inoculated with a multiplicity of infection 1 or 0.1 (MOI = 1 or 0.1) of SARS-CoV-2 isolate TGR/NY/20. At 72 h post-inoculation, cells were fixed and stained with a SARS-CoV-2 specific monoclonal antibody against the nucleoprotein [15]. Interestingly, no cytopathic effect nor immunofluorescence staining were observed in any of the infected cells. Vero E6/TMPRSS2 cells [36] were used as controls and demonstrated pronounced virus infection and replication.

### 3.2. Clinical Parameters and Assessment of Other Bovine Pathogens

**Figure 1:**
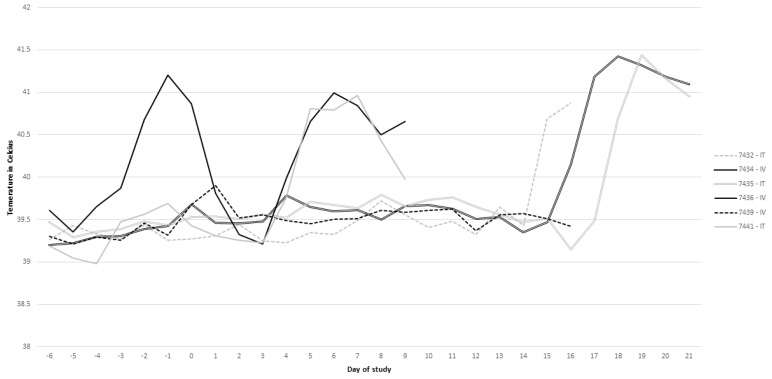
Rumen temperature measurements over time for each respective calf.

Clinical parameters were assessed following intratracheal (IT) and intravenous (IV) inoculation of SARS-CoV-2 in calves. Rumen temperature boluses recorded body temperature every 5 min and were averaged for each day over the course of the study. One calf (#7436) in the IV group presented a temperature >40 °C the day before inoculation, but the increase in temperature had resolved approximately 24 h after inoculation (Figure 1). All calves, excluding calf 7439 in the IV group, had a sustained temperature >40 °C for multiple days over the course of the study (Figure 1). The two calves (7436 and 7434) in the IV group presented increased body temperature on days 4–9 and 16–21, respectively (Figure 1). While calf 7439 in the IV group did not present body temperature >40 °C, this calf did have a slight increase in temperature immediately after inoculation (Figure 1). Calves 7441, 7432, and 7435 in the IT group presented increased temperature on days 4–9, 14–16, and 18–21, respectively (Figure 1). Right after inoculation, calves in the IV group exhibited open mouth and labored breathing in association with periodic coughing. These signs resolved within 15 min after inoculation and were most likely related to injection of inoculum via the IV route. Additionally, coughing was observed in the IT inoculated calves following inoculation but resolved within 5 min, which was attributed to the inoculation. Regardless of inoculation route, occasional coughing was observed in some animals 4–5 days following inoculation.

Nasal swabs collected prior to inoculation and at necropsy, as well as lung lavage, were submitted to the Iowa State Veterinary Diagnostic Lab for screening for routine bovine respiratory pathogens, including bovine viral (bovine herpesvirus-1 (BHV-1), bovine coronavirus (BCoV), bovine viral diarrhea virus (BVDV), and bovine respiratory syncytial virus (BRSV)) and bacterial pathogens (*Pasteurella multocida*, *Mycoplasma bovis*, *Mannheimia haemolytica*, and *Histophilus somni*). Samples tested negative for all bovine pathogens by PCR. Additionally, all animals were seronegative for viral targets BVDV, BRSV, BHV-1, bovine parainfluenza virus-3 (BPI-3), bovine adenovirus-3 (BADV-3), and BCoV using the BioX ELISA (BioX Diagnostics, Belgium, Europe) or a virus neutralization (VN) assay (BCoV).

### 3.3. Neutralizing Antibody Response to SARS-CoV-2 Infection

Serum was collected on days 0, 7, 14, and 21 to evaluate seroconversion to SARS-CoV-2 using a virus neutralization assay. Prior to the start of the study, all calves were seronegative to SARS-CoV-2 (<4). On day 7, all calves in the IT inoculated group presented neutralizing antibody (NA) titers of 8, and the IV calves presented NA titers of 4. Interestingly, these antibody titers were not sustained over the course of the 21-day experiment, and by day 14, calves in both the IT and IV group presented NA titers of 4 and two of the calves sampled on day 21 did not present detectable neutralizing antibody titers (<4).

### 3.4. SARS-CoV-2 Nucleic Acid Detection

Prior to the start of the study (day 0), no SARS-CoV-2 viral RNA was detected in any of the calves. SARS-CoV-2 RNA was detected (Ct < 40) in two nasal swab samples by rRT-PCR; calf (7441) in the IT group on day 3 and calf (7434) in the IV group on day 10 (Table 1). All other nasal and rectal swabs were negative (Ct > 40) for SARS-CoV-2 by rRT-PCR (Table 1). Additionally, urine and buffy coat samples collected from all calves tested negative for SARS-CoV-2 (Table 1). The positive nasal swab samples were confirmed positive by the National Veterinary Services Laboratory (NVSL)-United States Department of Agriculture (USDA), Animal and Plant Health Inspection Service (APHIS), Ames, IA. An extensive number of tissues were collected at necropsy and evaluated by rRT-PCR for SARS-CoV-2 (Table 2). Most tissues were negative, with the exception of the tracheobronchial lymph node of calf 7441 in the IT group that was necropsied on day 9. This calf also had a positive nasal swab on day 3 post-inoculation (pi). The positive samples were subjected to virus isolation and no infectious virus was detected in any of the samples.

### 3.5. Gross and Histologic Changes at Necropsy

At necropsy, minimal gross changes were observed. Regardless of day pi, gross changes were observed in kidney, consisting of multifocal small (1–3 mm) petechiae on the capsular surface of the kidneys. Additionally, the liver of calf 7434, necropsied on day 21 pi, was mottled, with multifocal small petechiae; the visceral surface was edematous, most notably in the area near the hepatic lymph node, which was enlarged and edematous. No notable changes were observed in other tissues collected at necropsy, as outlined in Table 2.

**Figure 2:**
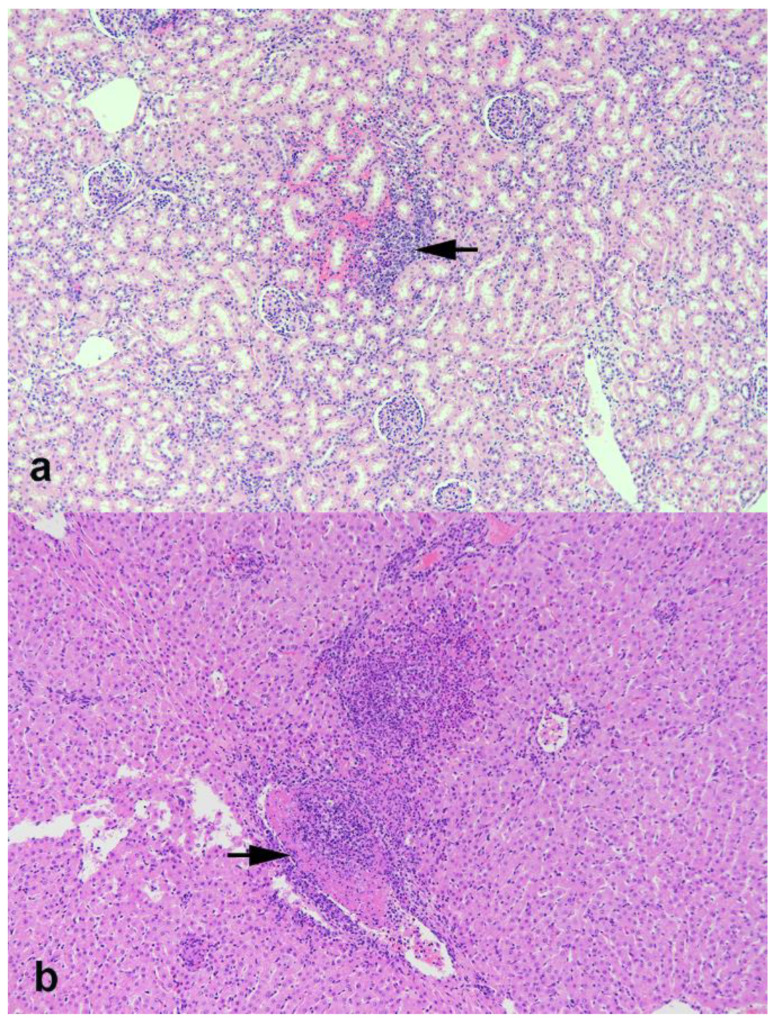
Photomicrographs of kidney (**a**) and liver (**b**) from calves experimentally inoculated with SARS-CoV-2. (**a**) Focal area of hemorrhage and infiltrates of lymphocytes and lesser numbers of neutrophils (arrow). (**b**) Multifocal area of necrosis with infiltrates of mixed inflammatory cells (neutrophils, macrophages, lymphocytes). Note vasculitis with fibrinocellular thrombus (arrow). Hematoxylin and eosin (HE). Magnification = 10×.

Histologically, the renal cortex contained multifocal small petechiae and infiltrates of lymphocytes and lesser numbers of neutrophils (Figure 2). The liver from calf 7434 in the IV treatment group contained multifocal areas of necrosis with infiltrates of mixed inflammatory cells (neutrophils, macrophages, lymphocytes) and occasional vasculitis with fibrinocellular thrombi (Figure 2). All other sections of tissues collected at necropsy, as outlined in Table 2, did not present remarkable histological changes.

### 3.6. In Situ Hybridization (ISH)

**Figure 3:**
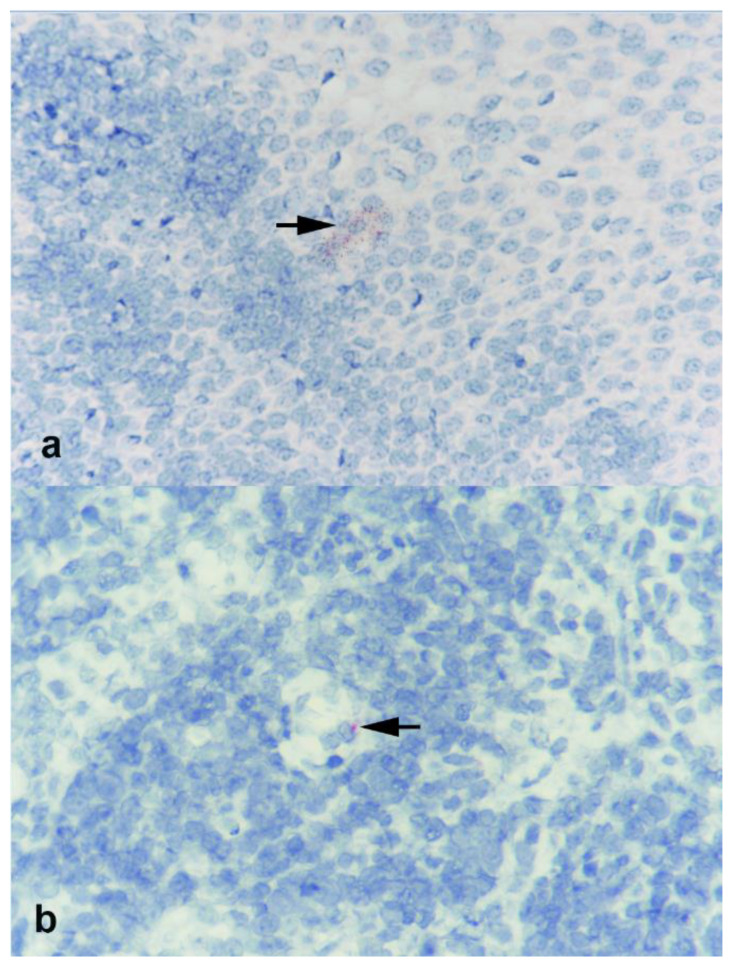
Photomicrographs of (**a**) palatine tonsil and (**b**) tracheobronchial lymph nodes from calf 7441 experimentally inoculated with SARS-CoV-2 and examined 9 days later. Sparse labeling for viral RNA is noted at base of stratified squamous epithelial layer in tonsil and in lymphoid follicle of lymph node (arrows). RNAscope ISH SARS-CoV-2. Magnification = 40×.

The tracheobronchial lymph node of calf 7441 in the IT group that was necropsied on day 9 pi and tested positive by rRT-PCR was evaluated by RNAscope for labeling of viral RNA (Figure 3). Sparse labeling was observed in the lymphoid follicle of the tracheobronchial lymph node of this animal. Additional tissues evaluated by RNAscope included the tonsil, kidney, nasal turbinates, lung, and trachea. Amongst those, sparse SARS-CoV-2 RNA labeling was only observed in the palatine tonsil of calf 7441 (Figure 3).

**Figure 4:**
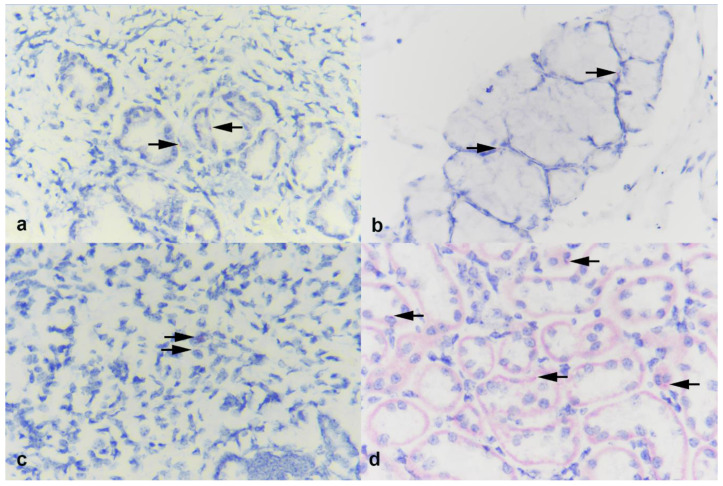
Photomicrographs of (**a**) turbinate, (**b**) palatine tonsil, (**c**) lung, and (**d**) kidney from calves experimentally inoculated with SARS-CoV-2 and examined 9 days later. In both turbinate and tonsil, sparse labeling for ACE2 receptor is seen, associated with submucosal glands (arrows). Sparse labeling is also noted in lung (arrows) and within renal tubular epithelial cells in the kidney (arrows). BaseScope ISH ACE2. Magnification = 40X.

Transcription of ACE2 was assessed in the turbinate, palatine tonsil, lung, and kidney from calf 7441 and 7434 by BaseScope (labeling of ACE2 mRNA) (Figure 4). In both the turbinate and tonsil, sparse labeling for ACE2 expression was seen associated with submucosal glands (Figure 4a,b). Sparse labeling was also noted in lung and to a greater degree within renal tubular epithelial cells of the kidney (Figure 4). While ACE2 expression is sparse in all calf tissues evaluated, slightly higher levels of ACE2 were observed in the kidney.

## 4. Discussion

The lack of productive replication in calves and lack of viral replication in bovine primary cells indicate that cattle are likely not susceptible to SARS-CoV-2 infection. This is supported by the fact that only two nasal swabs from two calves (7441 and 7434) and one tissue, the tracheobronchial lymph node from one calf (7441), tested positive for SARS-CoV-2 RNA by rRT-PCR. Importantly, only small amounts of viral RNA were detected in these samples and no infectious virus was recovered on virus isolation. Nominal labeling of SARS-CoV-2 by ISH in the tonsil and tracheobronchial lymph node of one calf highlights the low levels of virus RNA in these tissues and is consistent with the absence of viral replication. Baseline samples collected prior to inoculation were negative for both SARS-CoV-2 antigen and antibody and served as controls for the study.

The in vitro results showing no replication of SARS-CoV-2 in a panel of bovine cells support the in vivo results observed in this study. Furthermore, the low and transient levels of antibodies observed over the course of the study further support the conclusion of lack of SARS-CoV-2 replication in the calves inoculated here. These results are similar to other reports following intranasal inoculation of calves with SARS-CoV-2, in which two of six inoculated calves were positive by rRT-PCR (day 2 and 3 pi) in nasal swab samples and minimal seroconversion was also detected [26]. Despite three nasal swabs positive by rRT-PCR in the previous study, low viral genomic loads were detected, and this is further confirmed by the absence of transmission to direct contact calves [26].

Interestingly, while results here and those previously reported by Ulrich and collaborators [26] suggest lack of SARS-CoV-2 replication, a study utilizing ex vivo tracheal and lung organ explants of domestic ruminants supported sustained viral replication of SARS-CoV-2 [31]. Di Teodoro et al. also reported an association of SARS-CoV-2 replication with ACE2-expression in cells of the respiratory tract. The ACE2 labeling in the ex vivo tracheal and lung cultures was stronger [31] than what we observed directly in the lung of the calves in the current study. In the current study, the kidney presented the greatest expression of ACE2, followed by the sparse labeling in the turbinates and tonsil, with the lung only having minimal ACE2 expression when compared to the other tissues. These observations are similar to what has been described in other studies [32]. Differences in the degree of expression or labeling could be caused by several factors including the age of the animals, the ACE2 target, or the labeling technique between the current study and the ex vivo study [31]. With regard to the labeling technique and target, in the current study, the RNAscope^®^ probe targeted the mRNA translating amino acid (AA) region spanning AA 31–82, whereas the antibody used for immunohistochemistry (IHC) to detect labeling in the ex vivo cultures was targeted at AA 788–805, in the C-terminal of the ACE2 gene [31]. Additionally, the tissue from calves in the current study were approximately 9–10 weeks of age, whereas the age of calves used of the ex vivo cultures was 18 months.

Although no viral RNA was detected by rRT-PCR in the kidneys or the urine samples, the kidneys of all calves presented multifocal small petechiae on the capsular surface of the organ. Histological examination of the tissue confirmed the presence of multifocal hemorrhage and mild interstitial nephritis. Consistent with the rRT-PCR results, the RNAscope using the SARS-CoV-2 RNA probe confirmed the absence of virus in those lesions. The RNAscope was used to confirm rRT-PCR results given that acute kidney injury has been reported in COVID-19 patients and is one of the important complications associated with SARS-CoV-2 infection [37,38]. SARS-CoV-2 has not been detected in the kidneys, but the pathology associated with COVID-19 in the kidney may be induced by a cytokine storm-induced systemic inflammatory response [37,38]. It is possible that the lesions observed in the kidneys in the calves here may be due to an inflammatory response similar to that of sepsis-induced kidney injury. LPS-induced inflammation models are widely used as a method to induce systemic inflammation that mimics the initial clinical features of sepsis and results in acute kidney damage similar to that observed in the calves [39].

While the routes of inoculation utilized in the current study would not be considered likely routes of infection, given the previous reports in the literature [31,32], these routes were employed to exploit potential routes that would be most directly associated with tissues that have the greatest ACE2 receptor distribution in cattle. Immediately after inoculation, calves in the IV group exhibited transient clinical signs similar to those described in calves administered an LPS challenge IV [40]. Most calves, excluding one calf in the IV group, presented a sustained temperature >40 °C for multiple days over the course of the study. Given the lack of clinical signs, lack of detection of SARS-CoV-2, and of other typical bovine viral or bacterial pathogens in tissues and respiratory specimens, the increase in temperature is not supportive of pathogen-associated pyrexia. It is possible though that antigen-induced pro-inflammatory responses, following IV or IT inoculation of SARS-CoV-2, could have caused those manifestations. The study by Ulrich and collaborators [26], in which calves were inoculated intranasally with SARS-CoV-2, did not observe any clinical signs or changes following SARS-CoV-2 inoculation. The fact that we used colostrum-deprived immunologically naïve calves approximately 6 weeks of age and administered the virus IV or IT could also have contributed to these manifestations. Importantly, regardless of the differences in animal age, route of inoculation, and immune status of the calves used in the two studies, virological and serological findings were analogous between the studies. The consistency between the findings from Ulrich and collaborators [26] and our study suggest that cattle are not susceptible to SARS-CoV-2 infection and most likely may not function as a reservoir for the virus. While predictive analyses of the binding potential of SARS-CoV-2 with the ACE2 receptor suggests that cattle may be susceptible, the results from this study and previous reports suggest otherwise. This highlights the need to evaluate susceptibility to SARS-CoV-2 in vivo in the species of interest to determine the virus host range.

## Figures and Tables

**Table 1:** Summary of positive sample type and day as determined by rRT-PCR.

Route	Intratracheal Inoculation Group			Intravenous Inoculation Group		
Calf ID	7441	7432	7435	7436	7439	7434
Sample ^a^ day						
−3	-	-	-	-	-	-
−2	-	-	-	-	-	-
−1	-	-	-	-	-	-
0	-	-	-	-	-	-
1	-	-	-	-	-	-
2	-	-	-	-	-	-
3	+ *	-	-	-	-	-
4	-	-	-	-	-	-
5	-	-	-	-	-	-
6	-	-	-	-	-	-
7	-	-	-	-	-	-
8	-	-	-	-	-	-
9	-	-	-	-	-	-
10		-	-		-	+ *
11		-	-		-	-
12		-	-		-	-
13		-	-		-	-
14		-	-		-	-
15		-	-		-	-
16		-	-		-	-
17			-			-
18			-			-
19			-			-
20			-			-
21			-			-

^a^ For all animals samples tested included nasal swabs, rectal swabs, urine, and buffy coat. “-“ = not detected; “+” = detected, * Nasal swab, Ct value < 40. Grey shaded box highlights positive samples.

**Table 2:** Summary of samples and tissue collected at necropsy and rRT-PCR results.

	Intratracheal Inoculation Group			Intravenous Inoculation Group		
Calf ID	7441	7432	7435	7436	7439	7434
Necropsy day	9	16	21	9	16	21
adrenals	-	-		-	-	
bifurcation trachea	-	-	-	-	-	-
bronchi	-	-	-	-	-	-
bronchioles	-	-	-	-	-	-
cerebrum	-	-	-	-	-	-
colon	-	-	-	-	-	-
heart	-	-	-	-	-	-
hepatic LN						-
ICE junction	-	-		-	-	
kidney	-	-	-	-	-	-
left cerebellum	-	-	-	-	-	-
liver	-	-		-	-	-
lung	-	-	-	-	-	-
lung lesion	-	-	-	-	-	-
lung lavage	-	-	-	-	-	-
mediastinal LN		-	-			
mesenteric LN	-	-	-	-	-	-
mid trachea	-	-	-	-	-	-
muscle	-	--		-	-	
Peyer’s patch	-	-		-	-	
olfactory bulb			-			-
rectum	-	-		-	-	
retropharyngeal LN	-	-	-	-	-	-
right cerebellum	-	-	-	-	-	-
seminal vesicles	-			-		
sm/lg intestines	-	-		-	-	-
spleen	-	-		-	-	-
testicle	-	-		-	-	
thymus	-	-		-	-	
tonsil	-	-	-	-	-	-
tracheal wash	-	-	-	-	-	-
tracheobronchial LN	+	-	-	-	-	-
turbinates	-	-	-	-	-	-
urinary bladder	-	-		-	-	
vas deferens	-	-		-	-	

“-“ = not detected; “+” detected, Ct value < 40; black shaded box = no sample collected at necropsy; grey shaded box highlights positive sample.

## Data Availability

The data will be made available upon request via email to the corresponding author.
